# Epidemiological breast cancer prediction by country: A novel machine learning approach

**DOI:** 10.1371/journal.pone.0308905

**Published:** 2024-08-14

**Authors:** Hasna EL HAJI, Nada SBIHI, Bassma GUERMAH, Amine SOUADKA, Mounir GHOGHO

**Affiliations:** 1 TICLab, International University of Rabat, Rabat, Morocco; 2 Surgical Oncology Department, National Institute of Oncology, Mohammed V University, Rabat, Morocco; Hamadan University of Medical Sciences, School of Public Health, IRAN, ISLAMIC REPUBLIC OF

## Abstract

Breast cancer remains a significant contributor to cancer-related deaths among women globally. We seek for this study to examine the correlation between the incidence rates of breast cancer and newly identified risk factors. Additionally, we aim to utilize machine learning models to predict breast cancer incidence at a country level. Following an extensive review of the available literature, we have identified a range of recently studied risk factors associated with breast cancer. Subsequently, we gathered data on these factors and breast cancer incidence rates from numerous online sources encompassing 151 countries. To evaluate the relationship between these factors and breast cancer incidence, we assessed the normality of the data and conducted Spearman’s correlation test. Furthermore, we refined six regression models to forecast future breast cancer incidence rates. Our findings indicate that the incidence of breast cancer is most positively correlated with the average age of women in a country, as well as factors such as meat consumption, CO2 emissions, depression, sugar consumption, tobacco use, milk intake, mobile cells, alcohol consumption, pesticides, and oral contraceptive use. As for prediction, the CatBoost Regressor successfully predicted future breast cancer incidence with an R squared value of 0.84 ± 0.03. An increased incidence of breast cancer is mainly associated with dietary habits and lifestyle. Our findings and recommendations can serve as a baseline for developing educational programs intended to heighten awareness amongst women in countries with heightened risk.

## 1 Introduction

Breast cancer (BC) is the most prevalent cancer diagnosed in women (over a third of all female cancers). Even if cardiovascular diseases are the leading cause of death, it was estimated that deaths caused by cancer will exceed those caused by cardiovascular diseases in a few decades [1]. According to the World Health Organization, in 2020, there were 2.3 million women diagnosed with breast cancer and 685 000 deaths globally [2]. Besides physical and psychological suffering induced by breast cancer, all relatives may be affected, especially the children. Therefore, socio-economic consequences may be severe since two out of every three employees are forced to interrupt their careers [3].

In spite of high incidence rates, access to care and social support promotes health in developed countries [4]. Unlike in developing countries, where delays in diagnosis, high treatment costs, and a lack of support all contribute to increasing mortality rates [5, 6] and the situation worsened even further in the context of COVID-19, Morocco as an example [7, 8]. Thus, to take the first step toward BC prevention, it is crucial to understand the risk factors involved.

Despite the large number of breast cancer risk factors recently presented in the literature, the discussion and decision on the most relevant ones are still ambiguous. Expressly, the subject lacks a systematic literature review that identifies emerging risk factors and examines their association with the total incidence of breast cancer at a country level. It is particularly important to consider modifiable risk factors in order to provide some prevention recommendations. Education may be a fundamental part of this process, yet, only few programs focused on breast cancer education have emerged recently in this area [9]. Nonetheless, educational interventions for women at all phases of life are highly required. They may provide a good impact by raising awareness and encouraging self-care.

In addition to examining the emerging risk factors, it may also be important to find out their breakdown by country. It may even be more important to anticipate breast cancer future incidence rates per country. Some research studies have predicted the future incidence and mortality of breast cancer in some countries, such as Iran [10] Pakistan [11] and Japan [12]. Generally, they use time series models or they compute the number of new breast cancer incidence and deaths by multiplying the age-specific incidence (or mortality) rates estimated for a given year, by the corresponding expected population for a future interval of years. However, to the best of our knowledge, no study has been carried out in order to predict the incidence rate of breast cancer by country using machine learning and common risk factors shared between individuals in the same country.

Additionally, it should be highlighted that besides the investigated risk factors, it may also be relevant to incorporate some preventive reproductive factors, particularly breastfeeding and total fertility rates. We assumed that these factors could reduce the incidence rate of breast cancer since there is a growing body of research relating breast cancer risk reduction to breastfeeding [13]. Furthermore, we want to assess the association between fertility and country-specific breast cancer incidence.

The primary objective of this study is to review the most recent risk factors established through meta-analyses and systematic reviews. The secondary objective involves collecting and analyzing available data on risk and preventive factors from multiple countries, examining their correlation with reported breast cancer incidence rates. The third objective is to create a specific profile of modifiable risk factors that can be addressed within each country, such as external factors, population eating habits, and lifestyles. This analysis aims to propose potential solutions for mitigating the impact of these factors. Lastly, the study aims to develop a model that serves as a starting point for predicting future breast cancer incidence based on risk and preventive factors across countries.

The ultimate goal of this research is to conduct a larger study with the main objective of determining the current rates of each factor in each country and subsequently predicting future breast cancer incidence. This information can help identify high-risk countries and facilitate early detection strategies, particularly in developing nations where early detection programs are lacking, and cancer is often diagnosed at advanced stages. For example, if our model predicts a high future incidence rate in a particular country, this knowledge can assist the government in implementing appropriate actions, such as designing educational programs to raise awareness among the at-risk population.

## 2 Methods

Unlike traditional cancer studies that typically recruit cohorts, this study adopted an ecologic study design by gathering data at the country level. To predict breast cancer incidence, we examined the rates of risk factors across the entire population within each country. It is important to note that this study adheres to the guidelines outlined in Strengthening the Reporting of Observational Studies in Epidemiology (STROBE) [14].

Through an extensive bibliographic study, we examined the non-genetic risk factors associated with breast cancer. Subsequently, we collected data on these factors from various countries. Additionally, we included information on two preventive factors, namely the total fertility rate and breastfeeding rate. It is important to note that we deliberately excluded genetic factors from our analysis. Genetic factors pertain to individual-level data, which is not feasible when considering the overall population. Our focus was on factors that encompass the entire population rather than specific individuals.

In order to identify the most influential factors, we conducted correlation analyses between the risk factors and breast cancer (BC) incidence. It is worth noting that some of these factors lack consensus, and our study aims to establish their significance. Additionally, to forecast the future incidence of breast cancer on a country-by-country basis, we rigorously tested and refined six regression models.

### 2.1 Data collection

#### 2.1.1 Risk factors identification

In order to identify recent BC risk factors, we have conducted a systematic literature review on the related Meta-Analysis and Systematic Reviews published in 2021. Following the PRISMA protocol [15], we started the search process on February 2, 2022 by considering four digital libraries: Scopus, PubMed, Web of Science and Cochrane. The MeSH keyword used for the automatic search in the mentioned digital sources is “Breast Neoplasms”. The search strings used are: “Breast Neoplasms” AND “risk factors”. Furthermore, we have removed duplicates then we applied the following inclusion/exclusion criteria: *Inclusion criteria (IC)*

- IC: publications that include breast cancer non-genetic risk factors

*Exclusion criteria (EC)*

- EC1: studies not related to breast cancer and risk factors
- EC2: studies dealing with male breast cancer
- EC3: research articles that concern risk factors of breast cancer recurrence
- EC4: publications that treat genetic factors or family antecedent

#### 2.1.2 Construction of the dataset

In order to create our dataset, we gathered reported BC incidence rates dating back to 2018 in several countries, as well as available data reflecting the risk and preventive factors we were able to access. We extracted reported incidence rates of breast cancer (rates per 100 000 females in 2018, age between 0 and 84) from the Global Cancer Observatory [16], the average age of women per country from WorldData [17], the prevalence of insufficient physical activity, depression, overweight, obesity, BMI, alcohol consumption and smoking rates from the World Health Organization [18], CO2 emissions from the International Energy Agency [19], breastfeeding, contraception use and world fertility data from United Nations [20]. We extracted food supply quantities of meat, sugar and milk from the Food and Agriculture Organization [21], mobile cells from the OpenCellID [22] and surface area from the World Bank Open Data [23] for data normalization. Notably, we could not find data on the consumption of sugar-sweetened beverages, so we considered data on white sugar consumption by country since sugar is also known to be a risk factor for breast cancer [24].

### 2.2 Breast cancer incidence

Prior to conducting the analysis, we addressed missing values in our dataset by imputation. Specifically, we utilized the k-Nearest Neighbor algorithm (KNN) [25]. The KNN algorithm was applied individually to each feature with missing values, considering all other available features as input. During the imputation process, the KNN algorithm iterates through the dataset to identify “k” similar or closely related examples, also known as neighbors, based on spatial proximity. For each example with missing values, the algorithm imputes those missing values with the mean value derived from its k-neighbors. This method has been shown to effectively handle missing data and maintain the integrity of the dataset [25].

To gain a more comprehensive understanding of breast cancer incidence across countries, we categorized them into quartiles, which are subgroups that divide the countries based on their breast cancer incidence rates into four equal parts. Each quartile represents 25% of the total number of countries and is determined by the combination of three values that serve as thresholds for this division. This approach allows for a more nuanced assessment of how countries rank in terms of breast cancer incidence.

### 2.3 Correlation between breast cancer incidence rates and the studied factors

#### 2.3.1 Data normality assessment

To evaluate the normality of the distribution of each risk and preventive factor against the incidence of breast cancer, we employed both statistical and graphical methods. For each factor, we conducted the Shapiro-Wilk test [26] which is a statistical test that determines whether a sample comes from a normally distributed population. A p-value greater than 0.05 in the Shapiro-Wilk test indicates that the data do not significantly deviate from normality, suggesting a normal distribution. To support our findings, we also generated histograms and Q-Q (Quantile-Quantile) plots.

#### 2.3.2 Correlation analysis

Given the results of the normality assessments, we proceeded with the appropriate correlation analysis. The presence of non-normally distributed data necessitated the use of a non-parametric correlation test. Therefore, we opted for Spearman’s rank correlation coefficient [27], which measures the strength and direction of the association between two variables. For each risk and preventive factor, we calculated Spearman’s rank correlation coefficient with the incidence of breast cancer. This approach allowed us to identify significant associations while accounting for the non-normal distribution of our data.

### 2.4 Breast cancer incidence rate prediction

One of the purposes of this study is to anticipate breast cancer future incidence rate in each country using machine learning, specifically a regression model. In this section, we go through the prediction pipeline in great detail.

#### 2.4.1 Regression models

We have split the data into two sets, train with 67% and test with 33%. Then, we tested the following regression models:

- Linear Regression [28]
- Support Vector Regression (SVR) [29]
- Random Forest [30]
- 3 Gradient Boosting models based on decision trees: Catboost [31], XGBoost [32] and LightGBM [33]

Linear Regression [28] and Random Forest [30] are two fundamental regression models and boosting algorithms [34] are a class of ensemble learning where models are built sequentially so that each improves the error of the previous model. Catboost (Category Boosting) [31] applies gradient boosting on decision trees and achieve satisfactory results with no required parameter tuning. XGBoost (eXtreme Gradient Boosting) [32] is an ensemble model also based on parallel decision trees. By combining results from a set of simple and weak models, it generates a more accurate prediction. LightGBM (Light Gradient Boosted Machine) [33] is a gradient boosting framework that supports distributed learning, has a fast training phase and low memory usage and it provides efficient results. Finally, for SVR (Support Vector Regression) [29], typically Support Vector Machine (SVM) that is based on statistical learning theory can also perform as a regression method. Unlike classical regression models, where error is minimized by selecting the best hyperplane of fit, SVR sets a threshold error allowance around the regression hyperplane, ensuring that all data points within the threshold are not penalized.

#### 2.4.2 Evaluation metrics

In order to evaluate the models, we used:

- R squared (*R*^2^)
- Root Mean Squared Error (RMSE)
- Mean Absolute Error (MAE)

R-squared (*R*^2^) measures how closely the data points match the fitted regression hyperplane. Mean squared Error (MSE) is an absolute measure of the quality of the fit. It considers the sum of squares of error. Consequently, the square root of MSE is the Root Mean Squared Error (RMSE). Lastly, Mean Absolute Error (MAE) is similar to Mean Squared Error (MSE), however, it is calculated by adding the absolute values of error. MAE is a more straightforward depiction of the sum of error terms than MSE or RMSE. MSE penalizes high prediction errors by squaring them, whereas MAE handles all errors similarly.

#### 2.4.3 Features selection

We applied a Forward Feature Selection with cross-validation to each of the six regression models. Forward Feature Selection is an iterative approach for selecting features that resulted in good model performance. It starts with 0 features in the model and adds at each iteration the feature that best improves the model until adding a new variable does not enhance the model’s performance. Cross-validation (CV) is used to test the performance of a machine learning model and it is an efficient method when the data is limited since it generates different test sets. Afterward, we provided a comparison between the 6 models in terms of the above mentioned evaluation metrics. Finally, we selected the best regression model and retrained it on the selected features 50 times with different random splits of the training/test sets. The final result is the average performance over the 50 runs with their standard deviation.

## 3 Results

### 3.1 Data collection

**Fig 1:**
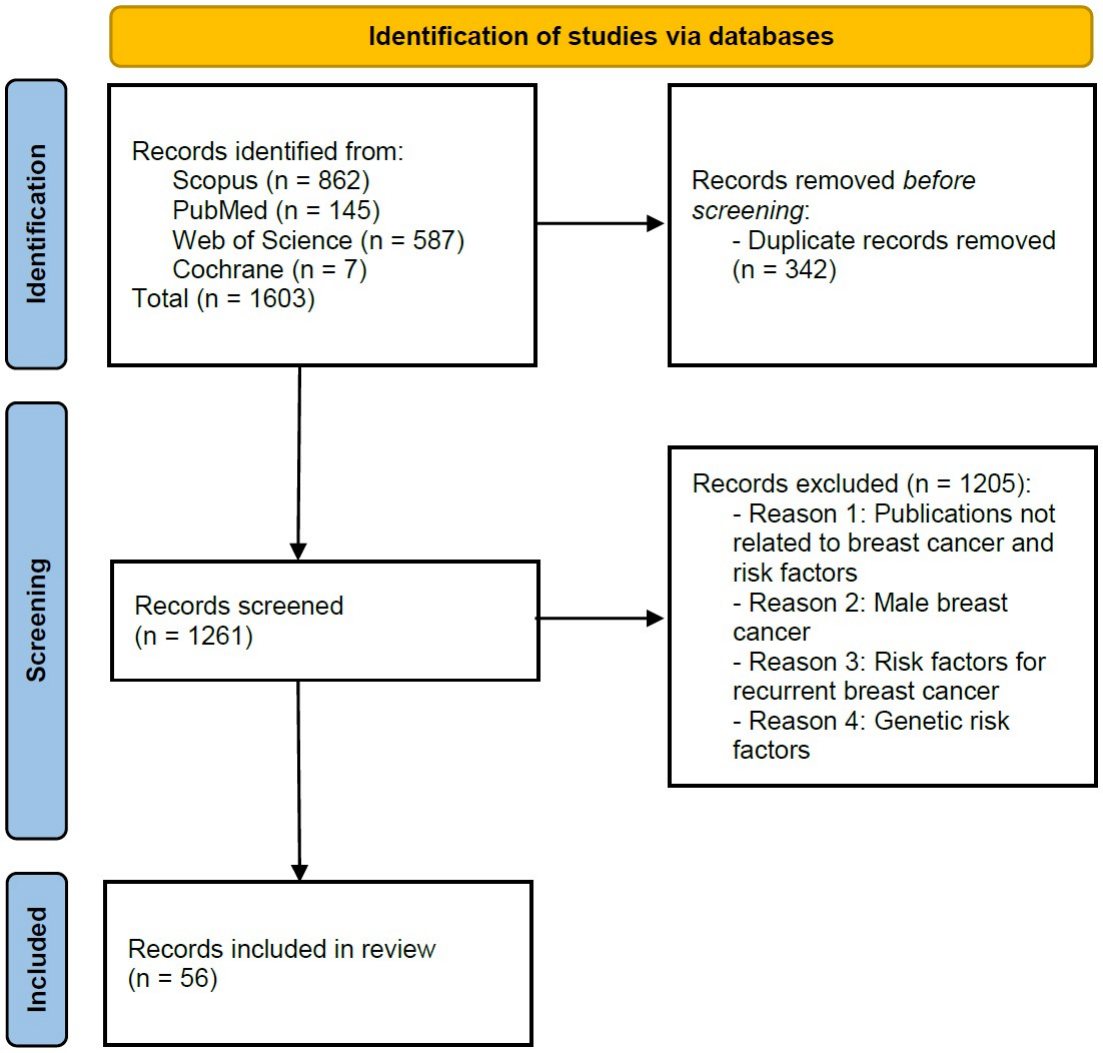
PRISMA flow diagram for articles’ screening and identification.

We have identified 1603 publications and then removed 342 duplicates. We conducted an analysis by inspecting each article’s title, abstract, and keywords. We identified a total of 56 studies after application of inclusion and exclusion criteria as presented in Fig 1. Accordingly, based on the full text analysis of the selected articles, we have categorized the non-genetic risk factors as presented in Table 1.

**Table 1:** Categorization of breast cancer risk factors identified from recent biomedical literature (2021).

Risk factor category	Risk factor designation	Reference
Hormonal factors	Oral contraceptives	[35–38]
	Menopausal hormone therapy	[39]
	Repercussions of melatonin	[40]
Reproductive factors	Early menarche	[41, 42]
Health and physical conditions	Insufficient physical activity	[43, 44]
	Long-term weight gain	[45]
	Obesity	[46–48]
	Chronic inflammation	[49]
	Type 2 diabetes	[48, 50]
	Vitamin D deficiency	[51]
	Breast skin microbiota	[52]
	Bariatric surgery	[53]
	Preeclampsia	[54]
	Thyroid disease	[55]
	Sleep-disordered breathing	[56]
Psychological Factors	Bipolar disorder	[57]
	Stress	[58, 59]
	Trauma, grief and depression	[60]
Lifestyle	Smoking	[61]
	Alcohol consumption	[62]
	Sedentary work	[63, 64]
	Night-shift work	[65, 66]
	Excessive Smartphone use	[67]
Eating habits	Pickled foods	[68]
	Consumption of red and processed meat	[69–71]
	Intake of Isoflavones	[72]
	Ultraprocessed food intake	[73]
	Consumption of sugar-sweetened beverages	[74, 75]
	Pro-inflammatory diet	[76, 77]
	Milk consumption	[69]
Medications	Aspirine use	[78, 79]
	Antihypertensive medication use	[80]
External exposures	Light at night exposure	[81, 82]
	Exposure to Polychlorinated Biphenyls	[83]
	Exposure to Endocrine disruptors	[84]
	Ambient air pollution exposure	[85]
	Pesticide exposure	[86]
	Hair chemicals	[87]
	Exposure to Polycyclic Aromatic Hydrocarbons	[88]
Demographic factors	Age (advancing age)	[89]
	Low socioeconomic status	[90]

In order to create the dataset, we collected reported BC incidence rates dating back to 2018 across 151 countries, along with data on 13 risk factors (available data on factors from Table 1) in addition to 2 preventive factors (breastfeeding and total fertility rates). We refer the reader to Appendix 1 for details about the extracted risk and preventive factors.

### 3.2 Breast cancer incidence

Based on breast cancer incidence rates reported in 2018 by the Global Cancer Observatory [16], our interpretation revealed that countries with very high levels of breast cancer incidence rates (ranging from 109.5 to 200.7) predominantly include the United States, Canada, Japan, Australia, and most European countries.

The second category with high level (48.5–109.5) includes Russian Federation, China, some North African countries (Morocco, Algeria and Tunisia), Indonesia, South Africa, two Eastern European countries (Belarus and Ukraine), Turkey in addition to Latin America except Mexico, Peru and Ecuador which belong to the third category.

The latter which is of medium incidence rate (24.60–48.5) encompasses mainly some Central Asian countries, India, the vast majority of Gulf countries, some Sub-Saharan African countries, namely Ethiopia, Kenya, Gabon, Cameroon, Nigeria and Namibia.

The last category with low incidence rate (4.4–24.60) aggregates the rest of Sub-Saharan African countries in addition to Pakistan, Afghanistan, Mongolia and Maldives.

### 3.3 Correlation between breast cancer incidence rates and the studied factors

#### 3.3.1 Data normality assessment

We conducted the Shapiro-Wilk test for each factor. The results of the test are displayed in Table 2. We also generated histograms and Q-Q plots (see Appendix 2) to visually inspect the data distribution for any deviations from normality. The histograms and Q-Q plots corroborate the results of the Shapiro-Wilk test, providing a consistent evaluation of data normality. Only depression and insufficient physical activity were normally distributed (P-values of 0.883 and 0.234 respectively according to Table 2). Therefore, we opted to use the Spearman’s correlation test to assess the correlation between the factors and breast cancer incidence rates.

**Table 2:** Normality test (Shapiro-Wilk) for each factor.

Factor	Statistic	P-value	Normal Distribution
CO2 emissions	0.73	< 0.05	False
Pesticides	0.42	< 0.05	False
Average age	0.94	< 0.05	False
**Depression**	0.99	0.883	True
Mobile cells	0.61	< 0.05	False
Alcohol consumption	0.96	< 0.05	False
Tobacco consumption	0.79	< 0.05	False
Sugar consumption	0.88	< 0.05	False
Meat consumption	0.94	< 0.05	False
Milk consumption	0.88	< 0.05	False
Obesity	0.97	< 0.05	False
**Insufficient physical activity**	0.98	0.234	True
Total Fertility Rate	0.89	< 0.05	False
Breastfeeding rate	0.96	< 0.05	False
Oral contraceptives	0.86	< 0.05	False

#### 3.3.2 Correlation analysis

Table 3 displays the results of the correlation test. The last column indicates the interpretation of Spearman’s correlation coefficient *ρ*. Six interpretations are possible [91]:

- Perfect association: |*ρ*| = 1
- Very strong: 0.80 ≤ |*ρ*| < 1
- Moderate: 0.60 ≤ |*ρ*| < 0.80
- Fair: 0.30 ≤ |*ρ*| < 0.60
- Poor: 0 < |*ρ*| < 0.30
- No association: *ρ* = 0

**Table 3:** Results of the Spearman’s correlation test (correlation between BC incidence rate and each factor).

Factor	Correlation	P-value	Interpretation
Average age	0.88	< 0.05	Very strong
Meat consumption	0.79	< 0.05	Moderate
CO2 emissions	0.71	< 0.05	Moderate
Depression	0.71	< 0.05	Moderate
Sugar consumption	0.71	< 0.05	Moderate
Tobacco consumption	0.69	< 0.05	Moderate
Milk consumption	0.64	< 0.05	Moderate
Mobile cells	0.63	< 0.05	Moderate
Alcohol consumption	0.57	< 0.05	Fair
Pesticides	0.56	< 0.05	Fair
Oral contraceptives	0.52	< 0.05	Fair
Insufficient physical activity	0.40	< 0.05	Fair
Obesity	0.36	< 0.05	Fair
Total Fertility Rate	-0.86	< 0.05	Very strong
Breastfeeding rate	-0.60	< 0.05	Moderate

All the factors are significantly associated with the incidence of breast cancer (*P* − *value* < 0.05) (Table 3). Concerning risk factors, average age is very strongly associated with BC incidence (correlations of 0.88). Meat consumption, CO2 emissions, depression, sugar intake, tobacco use, milk consumption, and mobile cells show moderate correlations with the occurrence of breast cancer (correlation of 0.79, 0.71, 0.71, 0.71, 0.69, 0.64 and 0.63 respectively). Alcohol consumption, pesticides, oral contraceptives, lack of physical activity, and obesity are all fairly linked to the incidence of the disease (0.57, 0.56, 0.52, 0.40 and 0.36 respectively). In terms of preventive factors, breast cancer incidence shows a very strong negative correlation with fertility (correlation of -0.86) and a moderate negative correlation with breastfeeding (correlation of -0.60). It makes sense that higher levels of risk factors are linked to an increased incidence of breast cancer, while higher levels of preventive factors are associated with a reduced incidence.

### 3.4 Breast cancer incidence rate prediction

Table 4 presents the results of prediction: comparing the performance of the 6 machine learning models in terms of R squared, RMSE and MAE.

**Table 4:** Evaluation results of regression models.

Regressor	Number of selected features	R squared	RMSE	MAE
**CatBoost Regressor**	13	**0.84 ± 0.03**	**20.39 ± 2.26**	**14.99 ± 1.62**
Light GBM Regressor	10	0.83 ± 0.04	21.24 ± 2.32	15.76 ± 1.66
Random Forest	10	0.83 ± 0.04	21.05 ± 2.55	15.23 ± 1.88
SVR	8	0.82 ± 0.03	22.11 ± 2.50	15.82 ± 1.70
XGBoost Regressor	15	0.81 ± 0.05	22.28 ± 3.06	16.06 ± 2.14
Linear Regression	2	0.78 ± 0.03	24.49 ± 2.31	19.27 ± 1.70

CatBoost Regressor achieves an R squared score of **0.84 ± 0.03** in predicting BC incidence rate, making it the best performing model against the other 5 regressors. Such value of R squared is considered high and the prediction is accurate as it shows that the model can predict well the incidence rate value for a given country. The result is confirmed by the RMSE and MAE metrics which recorded smaller values (20.39 ± 2.26 and 14.99 ± 1.62 respectively) for CatBoost Regressor compared to the 5 remaining models.

The Forward Feature Selection enabled the selection of 13 variables from a total of 15. The selected features are: CO2 emissions, pesticides, average age, mobile cells, tobacco consumption, sugar, milk and meat consumption, insufficient physical activity, obesity, breastfeeding, total fertility rate and oral contraceptives.

**Fig 2:**
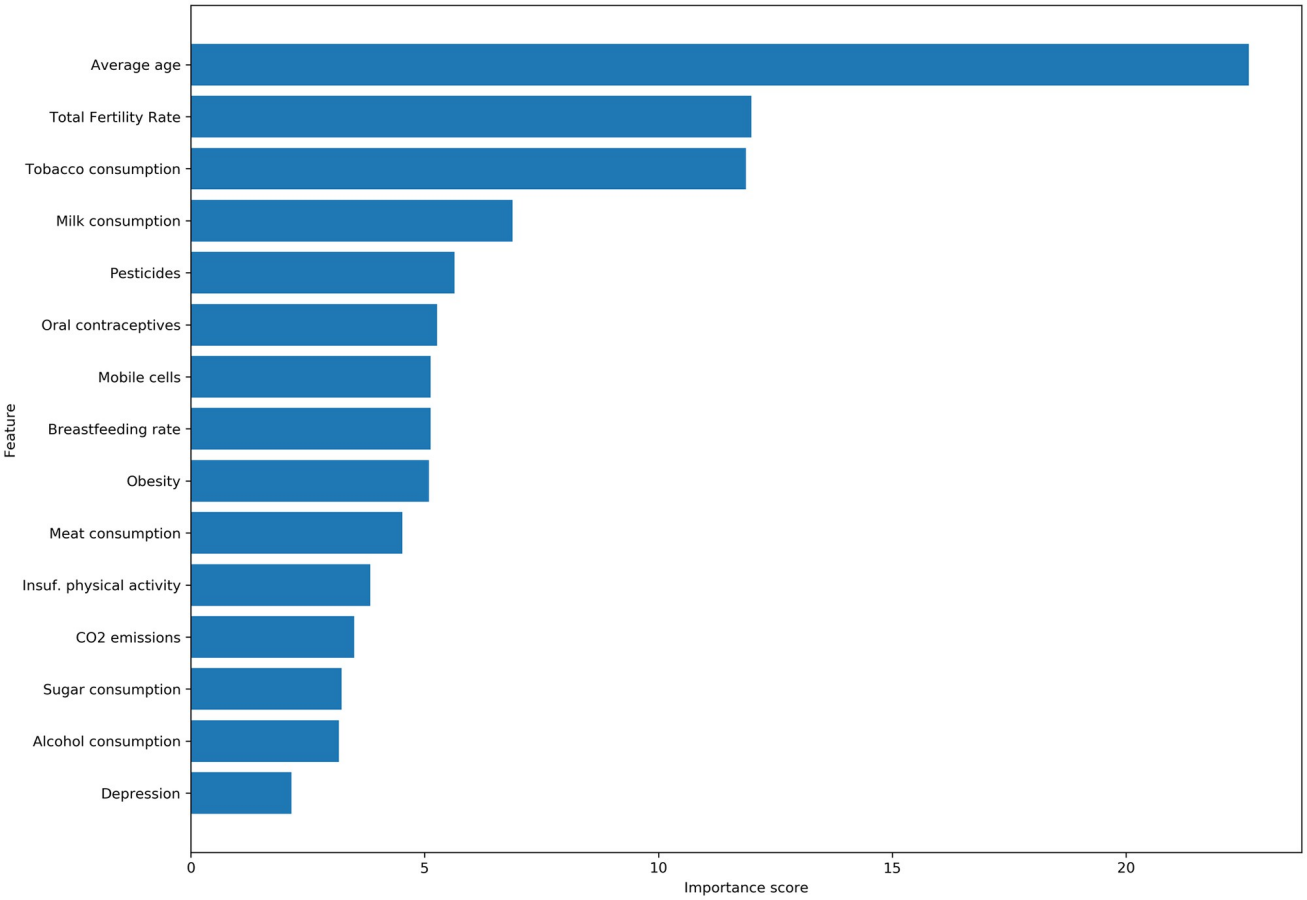
Feature importance using CatBoost.

The feature importance is computed using the impurity-based feature importance. Fig 2 represents the importance of each risk or preventive factor associated with breast cancer. The greater the score, the more important the feature. The importance of a feature is known as the Gini importance.

## 4 Discussion

Our study showed that age is the most correlated factor with breast cancer incidence (correlation of 0.88 according to Table 3). Other studies have confirmed that age is the most predominant risk factor for breast cancer [92], and the incidence rate increases significantly with age in different countries [93, 94]. Yet, younger women are not spared, for the latter, breast tumors are likely to appear in more complicated states, with positive lymph nodes, larger size and weaker survival [95].

According to our study, meat consumption is the most influencing eating habit positively correlated with the incidence of the disease (correlation of 0.79 according to Table 3). According to the International Agency for Research on Cancer (IARC), meat consumption can range from a few percent to 100% depending on the country [96]. In a recent research conducted using the Sister Study cohort [97], authors examined different types of meat and breast cancer incidence. They found that red meat consumption may increase the risk of invasive breast cancer [98]. The same conclusion has been drawn by several meta-analyses and systematic reviews insistently on processed meat [68–70].

Important correlation was observed regarding the association of breast cancer with CO2 emissions, depression and sugar consumption (0.71 according to Table 3).

A meta-analysis of 18 epidemiological studies indicates that exposure to ambient air pollution may have a significant impact on breast cancer development. Pooled analysis found that nitrogen dioxide exposure increases breast cancer risk [85]. As far as air particles are concerned, particulate matter PM2.5 and PM10 did not have significant associations with BC risk. Authors argue that further studies, particularly in developing countries, are needed to draw a firm conclusion of causality [85].

A recent retrospective cohort study [99] highlights a significant connection between depression and an increased risk of cancer, particularly breast cancer. The study demonstrated that individuals with depression showed an 18% overall increase in cancer diagnosis risk. This risk was most pronounced for breast cancer, with a hazard ratio (HR) of 1.23 (95% CI: 1.12–1.35, *p* < 0.0001) [99]. Additionally, we found that depression prevalence in a country as a psychiatric morbidity is associated with BC incidence. A recent systematic review investigating the impact of psychological factors on breast cancer risk has found that from twenty studies published between 1988 and 2020, only five reported a significant association between depression and BC incidence [60]. The authors emphasized that psychological factors deserve further investigation.

As for sugar consumption, the results of a study involving participants from a large prospective cohort confirmed our finding. Higher sugar intake with its different types has been associated with an increased risk of breast cancer [24]. Notwithstanding, the authors claimed that experimental data are needed to clarify the mechanisms behind these findings. Equally important, consumption of sugar-sweetened beverages may also increase the risk of breast cancer [74, 75].

Conforming to our study, tobacco consumption is the first lifestyle habit positively correlated with BC incidence (correlation of 0.69 according to Table 3). In this regard, a Mendelian Randomization was applied to investigate whether there is a causality between smoking and breast cancer risk [100]. The findings showed a possible causal relationship between lifelong smoking exposure and BC risk.

As for milk consumption, the correlation was 0.64 (Table 3). A study evaluating the association between dairy and soy milk consumption and BC risk joined us in concluding that higher consumption of dairy milk was linked to an increased risk of breast cancer [101]. In spite of this finding, a meta-analysis of 8 studies does not provide significant evidence that milk consumption is associated with breast cancer risk [102]. Accordingly, milk consumption is not consistently linked to breast cancer. To make a decision about this risk factor, further research is needed.

As for mobile cells, we considered the number of mobile cells per country divided by country area. We intended to evaluate the association between radio-frequency radiation exposure and BC prevalence. We found a moderate association with BC prevalence (correlation of 0.63 according to Table 3). This association was not examined in recent literature. Though, a Taiwanese case-control study has assessed the link between the behavior of Smartphone users and breast cancer risk (addiction, use before sleep, closeness to breast, etc) [103]. According to the study, excessive smartphone use increases the risk of breast cancer significantly.

Our results indicate that alcohol consumption is also positively correlated with BC incidence (correlation of 0.57 according to Table 3). The dose-response relationship between different types of alcohol and breast cancer risk was assessed in a meta-analysis [104]. The authors found a significant association between total drinking and breast cancer risk. The latter gradually increased with alcohol consumption, especially among postmenopausal women regardless of the kind of alcohol consumed.

Concerning pesticides, these are considered as endocrine disruptors likely to alter hormonal activity or cause epigenetic damage [105]. Our results indicate a correlation of 0.56 between BC incidence and pesticides (Table 3). A systematic review of 63 studies published between 1960 and 2019 showed that 62% indicated an association between pesticide exposure and BC, while 38% indicated the opposite. According to the authors, exposure to some types of pesticides may increase the risk of breast cancer [86].

Our study has found a fair link between oral contraceptives and breast cancer (0.52 according to Table 3), which is consistent with the findings of many recent studies [35–38]. The latter are all meta-analyses of case-control studies investigating the link between oral contraceptives and breast cancer. It was established that taking an oral contraceptive pill was linked to a considerably higher risk of breast cancer in totality. It’s important to note that the type of oral contraceptive, durability, dosage and age of starting use all have a part in this association.

Although obesity and insufficient physical activity are established risk factors for breast cancer [43, 44, 46–48], we could not uncover a strong link between these factors and the incidence of the disease since we found correlation of 0.36 and 0.40 respectively according to Table 3. Our result was also confirmed in a study rating BC risk factors [106]. Authors have demonstrated that obesity presents only a relatively modest risk for breast cancer, however, the risk of breast cancer is significantly increased by factors such as a genetic predisposition to the disease, a history of atypical hyperplasia and a history of neoplastic disease [106].

Regarding preventive factors, we observed a strong negative correlation between the total fertility rate and increased breast cancer risk (correlation of -0.86 according to Table 3). A prospective study conducted in Burkina Faso has provided supporting results [107]. The authors showed that, when comparing multiparous women to their non-multiparous counterparts, it was reported that multiparity decreases the risk of breast cancer [107]. This association is interesting and may be the subject of several observational studies to confirm or invalidate this point in other countries.

We also found that breastfeeding rate is moderately correlated with BC incidence (correlation of -0.60 according to Table 3). A systematic review and meta-analysis of studies published in the period of 1998–2021 uncovered a strong correlation between breastfeeding and risk of breast cancer [108]. The duration of breastfeeding was especially found to reduce BC risk [108].

A comparison between our findings and those of recent literature is given in Table 5. The table also provides some recommendations to reduce modifiable risk factors while emphasizing factors that need more research to determine their association with BC incidence.

**Table 5:** Comparison between our findings and those of recent literature and some recommendations to reduce modifiable risk factors.

Factor	Association assessed by other studies (at individual level)	Association assessed by our study (at country level)	Recommendations
Average age	Positive association [92–95]	Very strong association	Unfortunately, age is not a modifiable factor. However, we can suggest strengthening the screening in the countries with high levels of BC incidence
Tobacco consumption	Positive association [100]	Moderate positive association	To quit smoking, we propose to strengthen psychological therapies for assisting women by encouraging them to participate in free smoking cessation programs
Meat consumption	Positive association [68–70, 98]	Moderate positive association	These findings bolster public health recommendations to reduce meat consumption
Milk consumption	• Positive association [101]	Moderate positive association	More studies are needed on milk consumption to come to a definite conclusion
	• No significant association [102]		
Depression	Positive association [60, 99]	Moderate positive association	Screening for depression needs to become more sensitive
Alcohol consumption	Positive association [104]	Fair positive association	We join the 2020–2025 Dietary Guidelines for Americans. It is recommended that adults of legal drinking age may choose to abstain from alcohol consumption in order to reduce the risk of alcohol-related harms
Sugar consumption	Positive association [24]	Fair positive association	We insist on the American Heart Association recommendations. It suggests that, for women, added sugar should not exceed 100 calories a day (approximately 6 teaspoons) and also the consumption of sugar-sweetened beverages should be limited
Mobile cells	Positive association [103]	Fair positive association	The association between radiofrequency radiation exposure and BC incidence should receive more attention in future studies so that we can make recommendations accordingly
Oral contraceptives	Positive association [35–38]	Fair positive association	Generally, it may be beneficial to organize campaigns demonstrating the direct link between oral contraceptive use and breast cancer risk. Individually, every woman needs to discuss her contraception options with her physician.
CO2 emissions	Positive association [85]	Fair positive association	A recent literature review on ways to reduce carbon emissions from supply chains demonstrated the importance of coordinating with various means to reduce gas emissions. For instance how energy consumption is structured, production processes, and the optimal level of carbon emissions [110]
Pesticides	Positive association [86]	Poor positive association	Local fruits and vegetables are encouraged and they must be washed thoroughly before consumption.
Obesity	• Positive association [46–48]	Poor positive association	Even if we had not figure out a strong link between obesity and BC incidence, we recommend avoiding foods with high fat content and added sugar
	• No significant association [106]		
Insufficient physical activity	Positive association [43, 44]	Poor positive association	Our study found a weak link between insufficient physical activity and the prevalence of BC, yet we emphasize the current physical activity guidelines for Americans. In fact, women need 150 minutes of moderately intense exercise per week

In our view, there is a need for counseling about lifestyle habits (smoking and alcohol intake), as well as education about eating habits especially for those prone to other breast cancer risk factors, such as a family history of the disease. In fact, the most frequent risk factors responsible for BC onset are hereditary and genetic, such as breast cancer or ovarian cancer history and inherited mutations, in particular BRCA1 and BRCA2 [109].

As for the prediction, building an accurate model would highlight countries likely to register high incidence rates in the upcoming years. In terms of predictive ability, the CatBoost model yielded the best performance. It provided the most precise results when predicting breast cancer rates. In fact, a more precise regression is the one with a relatively high R squared (close to 1). For CatBoost Regressor, the average R squared is 0.84 ± 0.03.

CatBoost model was also used to identify factors that had a greater impact on breast cancer incidence prediction. Fig 2 shows that average age is the most significant predictive variable of breast cancer incidence. This is evident since the prevalence of BC increases after the age of 40 [93, 94], and a population with a median age higher than 40 is more likely to register a high incidence rate. Age is followed by total fertility rate and tobacco consumption (Fig 2).

### 4.1 Limitations and future directions

Our research has few limitations. First, given the lack of statistics regarding breast cancer, data were used from the majority of the developed countries but there was a lack of data concerning the United Kingdom. Also data from some Latin, Asian and African countries were not available. Nevertheless, this bias is frequently found in exploratory studies and can only be overcome if countries are more committed to communicating their data. Second, we did not consider genetic factors shared among individuals within the same population since related studies are heterogeneous and the only way would have been to do a meta-analysis on real aggregated data, which was not the scope of our study.

As a future direction, breast cancer incidence rates will be retrieved for various years and we will examine trends in breast cancer prevalence and related risk factors resulting from the present study. Our intention is to explore whether the changes in risk factors have influenced BC incidence rates.

## 5 Conclusion

Breast cancer is a complex disease influenced by multiple factors. Through our statistical analysis, we have identified several noteworthy associations. Specifically, we observed a significant positive correlation between the incidence of breast cancer in a country and the average age of women within that country, CO2 emissions, pesticides, depression rates, lifestyle, eating habits, mobile cells, and the use of oral contraceptives. Conversely, preventive factors such as breastfeeding rates and total fertility rates in a given country displayed a negative association with breast cancer prevalence. These findings informed the inclusion of these features in our breast cancer prediction model at the country level.

The prediction task was formulated as a regression problem, aiming to estimate the incidence rate of breast cancer. Our model demonstrated strong performance, achieving a mean R-squared score of 0.84 ± 0.03, underscoring the predictive power of our approach and the robustness of the regression model.

While this work provides valuable insights into predicting future breast cancer incidence rates and understanding major risk factors, it is important to interpret the results with caution due to the ecologic study design. Our findings are based on group-level data, and as such, cannot be directly translated to individual risk factors or causality. Nonetheless, this study highlights key areas for public health interventions and offers targeted recommendations for modifying certain modifiable factors, particularly lifestyle choices and eating habits. By addressing these factors at the population level, there is potential to make a substantial impact on reducing the burden of breast cancer.

## Supporting information

S1 Appendix (PDF)
