# Supplementary material for: Epidemiological breast cancer prediction by country: A novel machine learning approach
Source: PLoS One. 2024 Aug 14;19(8):e0308905. doi: 10.1371/journal.pone.0308905 (PMC11324133; doi:10.1371/journal.pone.0308905)
Supplement: S1 Appendix — (PDF) [file pone.0308905.s001.pdf]

Appendices

Appendix 1: Features description

In this section, we go over the risk and preventive factors that were considered, as well as their units:

- Average age: women average age per country
- Oral contraceptives: percentage of reproductive-age women who use oral contraception
- Insufficient physical activity : percentage of women attaining less than 150 minutes of moderate intensity physical activity per week, or less than 75 minutes of vigorous intensity physical activity per week, or equivalent
- Depression: number of women with depressive disorder (major depressive disorder/depressive episode or dysthymia). This involves symptoms such as depressed mood, loss of interest and enjoyment and decreased energy depending on the number and severity of symptoms. A depressive episode can be categorized as mild, moderate, or severe
- Obesity : percentage of defined population with a body mass index (BMI) of 30 kg/m2 or higher (Female)
- Sugar, meat & milk consumption: food supply quantity (unit: kg/capita/ year)
- Alcohol consumption: consumption of pure alcohol among people aged 15 years and older (unit: liters of pure alcohol per woman per year)
- Tobacco consumption : prevalence of current tobacco use between the ages of 15 and older (unit: percent)
- Breastfeeding rate : percentage of infants 0–5 months of age who are fed exclusively with breast milk
- Mobile cells: number of mobile cells per country divided by country area
- Total Fertility Rate : the mean number of children a woman would have by age 50 if she survived to age 50 and was subject, throughout her life, to the age-specific fertility rates observed in a given year. The total fertility is expressed as the number of children per woman
- CO2 Emissions: per capita carbon dioxide emissions by country
- Pesticides: Kg of pesticides per hectare of cropland divided by country cropland area

Appendix 2: Results of normality

Fig. 3 displays histograms and Q-Q plots for each of the factors examined.

Fig 3. Histogram and Q-Q (Quantile-Quantile) plot of each factor

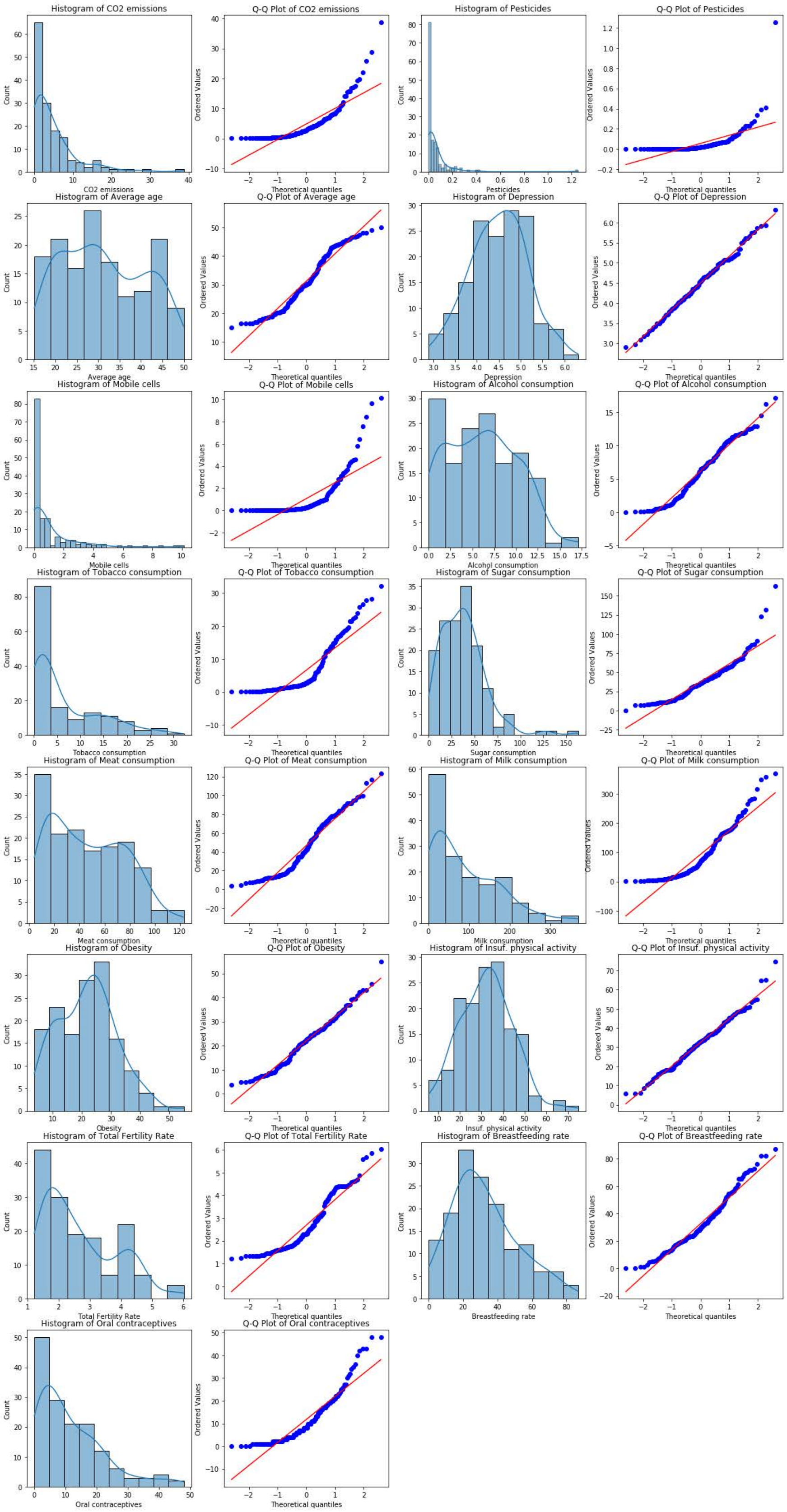
Histogram of CO2 emissions
Count
CO2 emissions
Q-Q Plot of CO2 emissions
Ordered Values
Theoretical quantiles
Histogram of Pesticides
Count
Pesticides
Q-Q Plot of Pesticides
Ordered Values
Theoretical quantiles
Histogram of Average age
Count
Average age
Q-Q Plot of Average age
Ordered Values
Theoretical quantiles
Histogram of Depression
Count
Depression
Q-Q Plot of Depression
Ordered Values
Theoretical quantiles
Histogram of Mobile cells
Count
Mobile cells
Q-Q Plot of Mobile cells
Ordered Values
Theoretical quantiles
Histogram of Alcohol consumption
Count
Alcohol consumption
Q-Q Plot of Alcohol consumption
Ordered Values
Theoretical quantiles
Histogram of Tobacco consumption
Count
Tobacco consumption
Q-Q Plot of Tobacco consumption
Ordered Values
Theoretical quantiles
Histogram of Sugar consumption
Count
Sugar consumption
Q-Q Plot of Sugar consumption
Ordered Values
Theoretical quantiles
Histogram of Meat consumption
Count
Meat consumption
Q-Q Plot of Meat consumption
Ordered Values
Theoretical quantiles
Histogram of Milk consumption
Count
Milk consumption
Q-Q Plot of Milk consumption
Ordered Values
Theoretical quantiles
Histogram of Obesity
Count
Obesity
Q-Q Plot of Obesity
Ordered Values
Theoretical quantiles
Histogram of Insuf. physical activity
Count
Insuf. physical activity
Q-Q Plot of Insuf. physical activity
Ordered Values
Theoretical quantiles
Histogram of Total Fertility Rate
Count
Total Fertility Rate
Q-Q Plot of Total Fertility Rate
Ordered Values
Theoretical quantiles
Histogram of Breastfeeding rate
Count
Breastfeeding rate
Q-Q Plot of Breastfeeding rate
Ordered Values
Theoretical quantiles
Histogram of Oral contraceptives
Count
Oral contraceptives
Q-Q Plot of Oral contraceptives
Ordered Values
Theoretical quantiles
